# Value of Clinical Information on Radiology Reports in Oncological Imaging

**DOI:** 10.3390/diagnostics12071594

**Published:** 2022-06-30

**Authors:** Felix Schön, Rebecca Sinzig, Felix Walther, Christoph Georg Radosa, Heiner Nebelung, Maria Eberlein-Gonska, Ralf-Thorsten Hoffmann, Jens-Peter Kühn, Sophia Freya Ulrike Blum

**Affiliations:** 1 Institute and Polyclinic for Diagnostic and Interventional Radiology, University Hospital Carl Gustav Carus Dresden, TU Dresden, 01307 Dresden, Germany; re.sinzig@gmx.de (R.S.); christoph.radosa@uniklinikum-dresden.de (C.G.R.); heiner.nebelung@uniklinikum-dresden.de (H.N.); ralf-thorsten.hoffmann@uniklinikum-dresden.de (R.-T.H.); jens-peter.kuehn@uniklinikum-dresden.de (J.-P.K.); sophia.blum@uniklinikum-dresden.de (S.F.U.B.); 2 Quality and Medical Risk Management, University Hospital Carl Gustav Carus Dresden, TU Dresden, 01307 Dresden, Germany; felix.walther@uniklinikum-dresden.de (F.W.); maria.eberlein-gonska@uniklinikum-dresden.de (M.E.-G.); 3 Center for Evidence-Based Healthcare, Medical Faculty Carl Gustav Carus Dresden, University Hospital Carl Gustav Carus Dresden, TU Dresden, 01307 Dresden, Germany

**Keywords:** oncological imaging, quality assurance, computed tomography, clinical information, radiological report

## Abstract

Radiological reporting errors have a direct negative impact on patient treatment. The purpose of this study was to investigate the contribution of clinical information (CI) in radiological reporting of oncological imaging and the dependence on the radiologists’ experience level (EL). Sixty-four patients with several types of carcinomas and twenty patients without tumors were enrolled. Computed tomography datasets acquired in primary or follow-up staging were independently analyzed by three radiologists (R) with different EL (R1: 15 years; R2: 10 years, R3: 1 year). Reading was initially performed without and 3 months later with CI. Overall, diagnostic accuracy and sensitivity for primary tumor detection increased significantly when receiving CI from 77% to 87%; *p* = 0.01 and 73% to 83%; *p* = 0.01, respectively. All radiologists benefitted from CI; R1: 85% vs. 92%, *p* = 0.15; R2: 77% vs. 83%, *p* = 0.33; R3: 70% vs. 86%, *p* = 0.02. Overall, diagnostic accuracy and sensitivity for detecting lymphogenous metastases increased from 80% to 85% (*p* = 0.13) and 42% to 56% (*p* = 0.13), for detection of hematogenous metastases from 85% to 86% (*p* = 0.61) and 46% to 60% (*p* = 0.15). Specificity remained stable (>90%). Thus, CI in oncological imaging seems to be essential for correct radiological reporting, especially for residents, and should be available for the radiologist whenever possible.

## 1. Introduction

Correct diagnosis is particularly important in radiological imaging. Radiological reports containing errors could lead to negative consequences for the patient. Especially in tumor patients, findings in computed tomography (CT) represent an important component in clinical care. Possible detrimental effects include treatment discontinuation, withdrawal from a clinical study, or from biopsies of newly identified lesions for further clarification [1]. Overall, the subsequent therapy is decisively shaped and influenced by radiological findings.

Many prerequisites are needed for high reading accuracy. In addition to the image quality itself, professional experience could also have an impact on the error rate in radiological interpretation [2,3,4].

In the context of steadily increasing CT examination numbers, the radiologist is more and more exposed to a large amount of image data. Usually, the radiologist must read the CT data sets, identify the lesions, evaluate them correctly, and finally write the radiological report. Obviously, screening this amount of data, paired with the hecticness of routine practice and often missing or incomplete clinical information is prone to false assessments. In addition, radiological reports are often, but possibly falsely, considered to be objective and definitive.

Multiple studies show a positive impact on interpretation accuracy when clinical information is available for radiologists [5]. In most cases, an additional benefit of clinical information was shown for emergency diagnostics (stroke, abdominal pain, fractures, etc.) [6,7,8]. Investigations on the effect of clinical information on the reading accuracy of oncological imaging only provide an overview, and relevance for certain disease entities is unknown.

Imaging of oncological patients frequently accounts for a considerable portion of the workload in radiology departments [9]. In the context of population growth combined with increasing age, new cancer diagnoses may globally increase from 18.1 million in 2018 to 29.4 million cases in 2040 [10]. This clearly leads to a further growing number of oncological patients and to a larger number of radiological examinations. Precise radiological reports will therefore continue to be pivotal in the care of oncological patients.

Therefore, the purpose of the present study was to investigate the contribution of clinical information to the interpretation accuracy of radiologists in the setting of primary and follow-up oncological CT imaging. Furthermore, the impact of the radiologists’ experience levels on the interpretation accuracy of different tumor entities was analyzed.

## 2. Materials and Methods

### 2.1. Study Design and Imaging Protocol

The present study was approved by the local ethics committee (EK 416092019) and conforms to the Declaration of Helsinki. Written consent by the patients was waived because of the lack of clinical consistency and the conduct of the study in a retrospective design.

Data for study inclusion were acquired from the hospital information system. CT data sets of patients with proven tumor disease were obtained with the clinical indication “primary tumor diagnosis”, comprising the following entities: bronchial carcinoma, gastroesophageal carcinoma, carcinoma of the bladder, renal cancer, hepatocellular carcinoma, pancreatic cancer, and lymphoma. All cancer patients received a thoracoabdominal CT as part of primary staging or follow-up imaging in a clinical setting. Other techniques such as PET, MRI, and SPECT were not included as they do not represent the very initial staging examination and they are rather induced by the first CT. In addition, the study cohort was supplemented by a control group consisting of CT examinations without tumor diagnosis. The initial indication for CT in the cancer-free patients was either clinical tumor suspicion, which was ultimately not confirmed, or tumor follow-up after completion of therapy. Patients with conspicuously altered anatomy, such as condition after hemicolectomy or hemihepatectomy, were excluded. After study inclusion, tumor diagnosis, age, and sex were documented for every patient.

The CTs were performed on a 64-slice CT scanner (Siemens Somatom Definition AS+/Edge and Siemens Somatom Force; Siemens Healthineers, Erlangen, Germany) and included a postcontrast examination with the following protocols: 120 kV; intravenous application of 1.0–1.5 mL/kg iodinated contrast agent (Ultravist^®^ 370; Bayer Vital GmbH, Leverkusen, Germany), 35 s and/or 70 s delay; primary axial reconstructions in 3.0 mm-slice thickness. All examinations were performed from 06/2014 to 11/2019. The included patients were randomly registered for the present study.

### 2.2. Definition of the Gold Standard

Sixty-four tumor patients and twenty patients without active tumor disease were consecutively enrolled. An independent study coordinator defined a gold standard for each patient (primary tumor/no tumor, hematogenous and lymphogenous metastases) using follow-up examinations and pathology reports. The primary tumors and hematogenous/lymphogenous metastases had to be clearly delineated on CT. Additionally, metastasis either had to be confirmed by hybrid imaging in the further course, in a follow-up CT imaging by growth/response to therapy, or by histology (biopsy/surgery). Confirmed lymphogenous metastases had to be at least 1 cm in short-axis diameter to be classified as malignant.

### 2.3. Review Protocol

Three radiologists with different levels of experience independently reviewed the CT datasets. Two of them were experienced senior radiologists (R1: 15 years, R2: 10 years of CT imaging experience); the third reader was a radiology resident (R3: 1 year of CT imaging experience). Prior to the review, CT datasets were anonymized and stored in separate folders on a secured server. The readers were always blinded to the distribution of tumor vs. tumor-free patients. Each reader initially examined the patients’ CT datasets without any clinical information. With an interval of at least 3 months, the datasets were presented again in a varied order with details of patient history. The aim was to reflect everyday clinical practice as closely as possible. We only used the clinical information that was provided in the request for the CT scan. However, suspected diagnoses were excluded.

Readers had to detect a primary tumor and hematogenous/lymphogenous metastases. Their results were recorded in each round. The evaluation was based on a finding matrix on which the individual organ regions were listed. In case of an absent primary tumor or metastases, the reader’s decision was noted accordingly. Possible benign differential diagnoses were not documented.

### 2.4. Statistical Analysis

Statistical analyses were performed with SPSS (Version 28; IBM, Armonk, New York, NY, USA) and Excel 2016 (Microsoft Corporation, Redmond, Washington, DC, USA). Patient demographics (sex, tumor entities, and metastases) were indicated as absolute and relative frequencies. Sex was given as absolute and relative value. Patient age was checked for normal distribution using a Kolmogorov–Smirnov test. This variable was expressed as the median and interquartile range (IQR). For demographic comparisons (age and sex) the Mann–Whitney U test was applied. After reading completion, the data were compared with the gold standard to calculate true positive/negative and false positive/negative values for all readings. Accuracy, sensitivity, specificity, and positive/negative predictive values (PPV/NPV) for primary tumor and hematogenous/lymphogenous metastasis detection were calculated. Overall values were calculated as the sum of the documented reading results from all three radiologists. If a single patient had metastases from two or more regions, only the detection of all metastases was considered correct. To compare accuracies, sensitivities, and specificities, the chi-square and Fisher’s exact test were applied. Interrater agreement was calculated with Fleiss’ kappa statistic. The significance level was set at α < 0.05.

## 3. Results

Selected preliminary results have been presented in abstract/poster format at the German Congress of Radiology 2021 and European Congress of Radiology 2022.

A total of 84 patients were included in the present retrospective single-center study. Of these, 64 patients (49 men and 15 women; median age 64.5, IQR = 58–72 years) with known active cancer disease and 20 patients (12 men and 8 women; median age 58.5, IQR = 41–68.25 years) without an active cancer disease were enrolled. Patient age was not normally distributed (*p* = 0.03). Table 1 shows the demographics of the study population.

Table 2 shows the different tumor entities and locations of lymphogenous and hematogenous metastases. There was an equal distribution of primary tumors. Lymph node metastases were present in 19 patients (3 patients with two regions), and hematogenous metastases in 16 patients (2 patients with two regions).

Given three readers and 84 patients, 252 total radiology reports were assessed. Thus, there were 192 with primary tumor and 60 without tumor diagnosis, 57 with and 195 without lymph node metastases, 48 with and 204 without hematogenous metastases.

Overall, diagnostic accuracy for detecting primary tumor increased significantly from 77% (195/252) without clinical history to 87% (219/252), when clinical information was available (*p* = 0.01). Sensitivity of primary tumor detection increased significantly upon receiving clinical information from 73% (140/192) up to 83% (160/192; *p* = 0.01). In the subanalysis all readers performed better when clinical information was given, especially the less experienced reader with significant improvement [R1: 84% (54/64) vs. 91% (58/64), *p* = 0.28; R2: 70% (45/64) vs. 78% (50/64), *p* = 0.31; R3: 64% (41/64) vs. 81% (52/64), *p* = 0.01]. Specificity also increased overall when providing clinical information from 92% (55/60) up to 98% (59/60; *p* = 0.15), as well as for two out of three readers [R1: 85% (17/20) vs. 95% (19/20), *p* = 0.6; R2: 100% (20/20) vs. 100% (20/20), *p* = 1.00; R3: 95% (19/20) vs. 100% (20/20); *p* = 0.49]. Overall, PPV increased from 97% to 99% (R1: 95% vs. 98%; R2: 100% vs. 100%; R3: 98% vs. 100%) and NPV increased from 52% to 65% (R1: 63% vs. 76%; R2: 51% vs. 59%; R3: 45% vs. 63%) for primary tumor detection when patient history was available. The interrater reliability increased with available clinical information from fair to moderate (Fleiss’ kappa 0.26 vs. 0.41).

**Figure 1:**
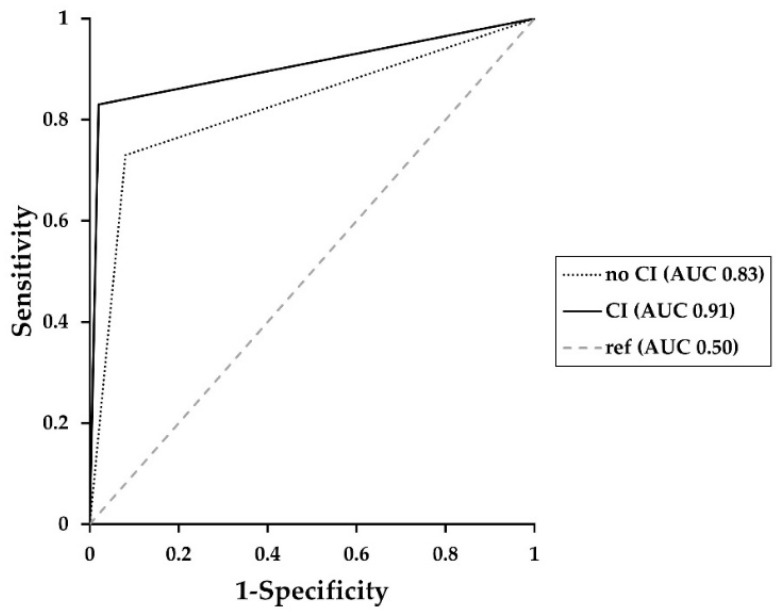
Receiver operating characteristic curves (ROC) showing the diagnostic performance of primary tumor detection over all radiologists whether clinical information (CI) was provided. Area under the curve (AUC) without CI = 0.83; with CI = 0.91. See Appendix A for ROC and AUC values for each reader.

Table 3 shows accuracy, sensitivity, and specificity for primary tumor detection. For receiver operating characteristic curves (ROC) see Figure 1.

A detailed analysis of the different tumor entities revealed heterogenous results (see Table 4). In bronchial carcinoma, all readers achieved a major improvement when clinical information was provided (63% vs. 89%; *p* = 0.05). In contrast, a decrease was found in hepatocellular carcinomas (74% vs. 70%; *p* = 1.00).

Table 5 depicts the results for primary tumor detection compared with selected studies. For most entities, similar sensitivities were found but in bronchial and lymphoma a difference of 9% and 19% occurred.

**Figure 2:**
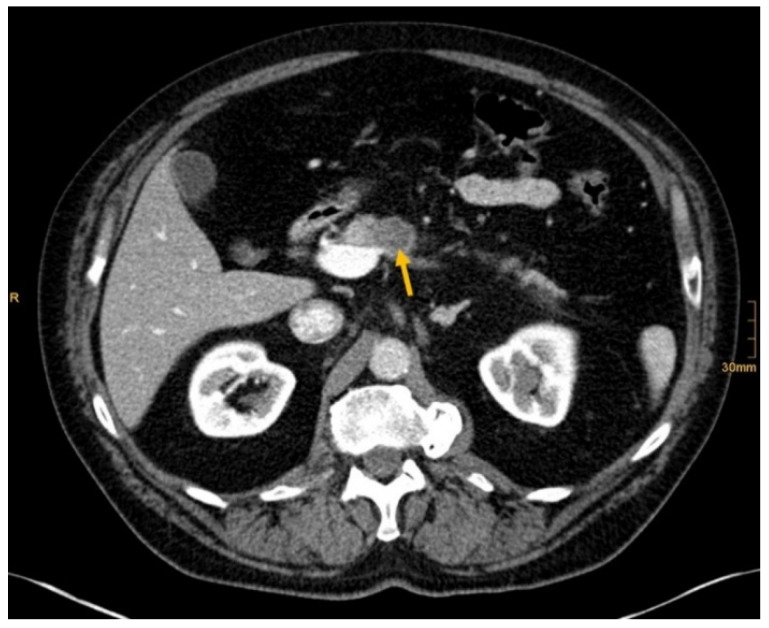
Pancreatic cancer (arrow). Correctly identified by one radiologist without and by all three radiologists with given clinical information (Conspicuous pancreas lesion seen in abdominal ultrasound. Laryngeal carcinoma several years ago.).

**Figure 3:**
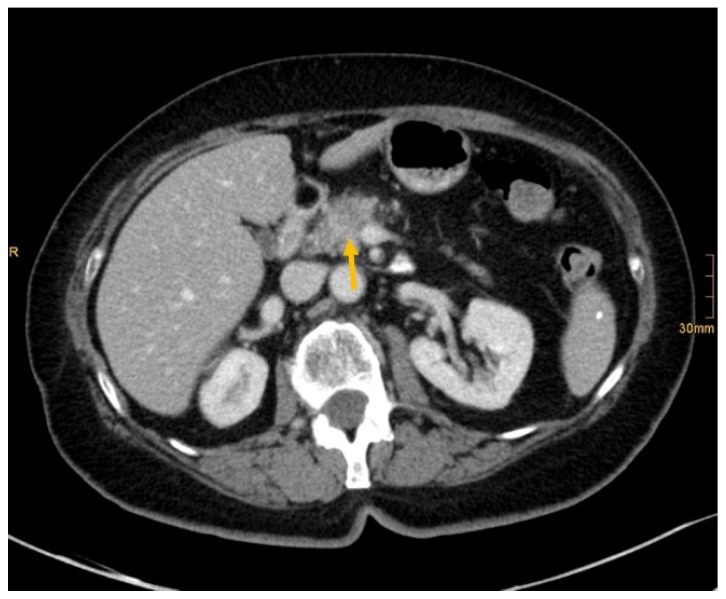
Pancreatic cancer (arrow). All readers identified the lesion in absence and presence of clinical information (Recurrent epigastric pressure and 9 kg weight loss in 4 months.).

**Figure 4:**
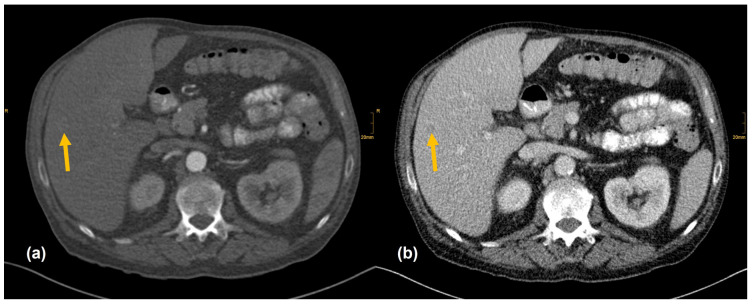
Hepatocelluar carcinoma in the right lobe (arrow). (**a**) Low contrast enhancement in the arterial phase. (**b**) Washout in the venous phase. All readers did not report the nodule whether clinical information was provided (Seizure after alcohol withdrawal. Known liver cirrhosis and hepatosplenomegaly.).

**Figure 5:**
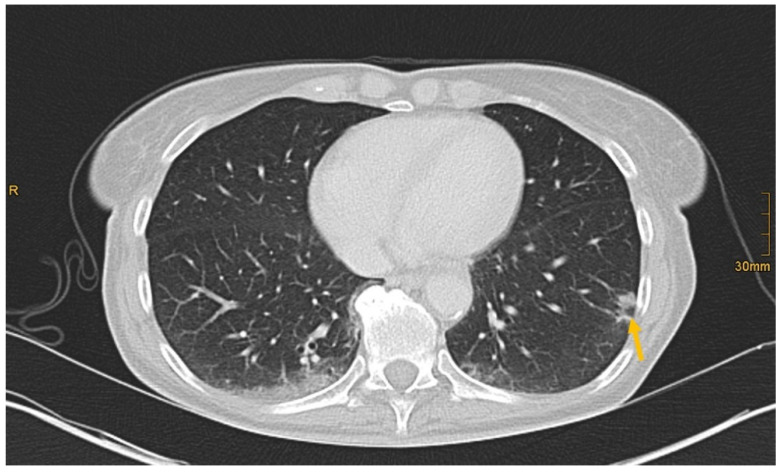
Subpleural bronchial carcinoma in the left lower lobe (arrow). Correctly identified by one radiologist without and by all three radiologists with given clinical information (Pain left hip with radiation into thoracic wall. Limited mobilization, reduced appetite. Obscure lesion of the lung on previous imaging.).

Figure 2, Figure 3, Figure 4 and Figure 5 show examples of primary tumor detection with detailed information on the reading results.

Diagnostic accuracy for detecting lymph node metastasis increased from 80% (202/252) to 85% (215/252) when clinical history was available (*p* = 0.13). For all readers, sensitivity of lymph node metastasis detection increased from 42% (24/57) to 56% (32/57) upon receiving clinical information (*p* = 0.13). The separate evaluation of the radiologists showed the largest increase in sensitivity for the less experienced reader, whereas the most experienced reader showed no change of sensitivity [R1: 26% (5/19) vs. 26% (5/19), *p* = 1.00; R2: 58% (11/19) vs. 68% (13/19), *p* = 0.73; R3: 42% (8/19) vs. 74% (14/19), *p* = 0.10]. Overall, specificity of lymph node metastasis detection increased when providing clinical information from 91% (178/195) to 94% (183/195; *p* = 0.33), as well as for two out of three readers [R1: 91% (59/65) vs. 98% (64/65), *p* = 0.11; R2: 91% (59/65) vs. 94% (61/65), *p* = 0.74; R3: 92% (60/65) vs. 89% (58/65), *p* = 0.76]. Overall, PPV increased from 59% to 73% (R1: 45% vs. 83%; R2: 65% vs. 76%; R3: 62% vs. 67%) and NPV increased from 84% to 88% (R1: 81% vs. 82%; R2: 88% vs. 91%; R3: 85% vs. 92%) for lymphogenous metastasis detection when providing clinical data. The interrater reliability decreased with available clinical information from moderate to fair (Fleiss’ kappa 0.42 vs. 0.29).

Diagnostic accuracy for detecting hematogenous metastasis did not relevantly change when clinical history was available [85% (213/252) vs. 86% (217/252), *p* = 0.61]. Overall, sensitivity for hematogenous metastasis detection increased from 48% (23/49) up to 60% (29/48) upon receiving clinical information (*p* = 0.15). The separate evaluation of the radiologists showed the highest increase in sensitivity for the less experienced reader, whereas the most experienced reader’s sensitivity did not change [R1: 56% (9/16) vs. 56% (9/16), *p* = 1.00, R2: 56% (9/16) vs. 63% (10/16), *p* = 1.00, R3: 31% vs. 63%, *p* = 0.16]. Overall, specificity of hematogenous metastasis detection decreased when providing clinical information from 93% (190/204) to 92% (188/204; *p* = 0.70). Although specificity of the less experienced reader dropped, the others did not change or increased moderately [R1: 96% (65/68) vs. 96% (65/68), *p* = 1.00; R2: 93% (63/68) vs. 94% (64/68), *p* = 1.00; R3: 91% (62/68) vs. 87% (59/68), *p* = 0.58]. Overall, PPV increased from 62% to 64% (R1: 75% vs. 75%; R2: 64% vs. 71%; R3: 45% vs. 53%) and NPV increased from 88% to 91% (R1: 90% vs. 90%; R2: 90% vs. 91%; R3: 85% vs. 91%) for hematogenous metastasis detection when providing clinical data. The interrater agreement decreased with available clinical information from moderate to fair (Fleiss’ kappa 0.41 vs. 0.32).

**Figure 6:**
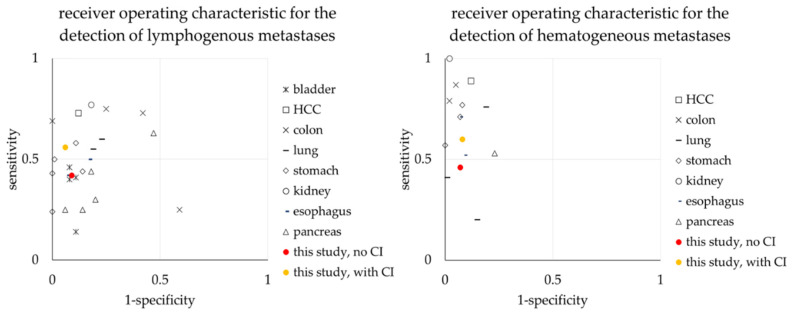
Comparison of sensitivities and specificities for metastasis detection per entity vs. reported data in the literature [18,19,20,21,22,23,24,25,26,27,28,29,30,31,32,33,34,35,36,37,38,39,40]. Lymph node metastasis on the left, hematogenous metastasis on the right.

For a literature comparison of sensitivity and specificity for metastasis detection see Figure 6. For true and false positive/negative findings for primary tumor and metastasis detection per reader see Appendix B.

## 4. Discussion

In our study, we investigated the value of clinical information on radiology reports in the diagnosis of different tumor entities and metastases and, in addition, their dependence on the level of radiologists’ experience. Our study is the first to show the positive impact of clinical information in oncological imaging.

Overall, we demonstrated the benefit of clinical information on the accuracy of oncological CT imaging with an increase of 10% in primary tumor, 5% in lymphogenous, and 1% in hematogenous metastasis detection. Loy et al. and Castillo et al. conclude that the presence of clinical data leads to higher diagnostic accuracy across all imaging modalities (e.g., radiographs, CT, MRI) and different clinical questions [5,41]. However, so far there are mainly studies on emergency imaging [5,6,7,8]. In literature, substantial increases in accuracy are partially reported (47% vs. 58% for detecting acute strokes in CT), but also a moderate advance is possible (79% vs. 82% in conventional radiography diagnosis of foot fractures) [6,8]. Oncological CT imaging thus enters the current studies with a substantial positive effect of clinical information on radiological reporting.

The positive effect of clinical information on radiological findings is also evident when considering sensitivity in the diagnosis of primary tumors. Overall, clinical information caused a significant increase of 10% in sensitivity in primary tumor detection, also confirming prior reviews [5,41]. We also found a positive impact of clinical information on sensitivity for the different tumor entities (Table 4), except for renal carcinoma (no change) and hepatocellular carcinoma (minor decrease). Previous studies frequently describe similar primary tumor detection rates to our data (Table 5) [11,12,13,14,15,16,17]. Only lymphoma and bronchial carcinoma diagnoses were inferior to comparable studies. It should be noted that different tumor stages could not be compared. Furthermore, the studies listed in Table 5 focused on one tumor type and not on several different primary tumors as we did. In addition, the available diagnostic options were mostly fixed to the examined tumor entity (e.g., the presence of a bronchial carcinoma). In the present study, there were no restrictions on tumor entities. Therefore, we assume that our study represents a realistic view of everyday clinical practice.

We recorded a benefit in metastasis detection when patient history was available. For both hematogenous and lymphogenous metastases, the percental increase in sensitivity was greater (14% and 12%, respectively) than for primary tumor detection (10%). However, the absolute improvement was low given the small number of metastases, and overall, the sensitivity of metastasis detection was considerably below that for primary tumors. Merely, Çiray et al. investigated the influence of additive clinical information on metastasis diagnosis. They found an increasing rate of true positive and true negative results for malignancy in CT imaging of bone metastases from 44% up to 82% when clinical information was provided [42]. Literature provides heterogeneous results regarding detection rates of metastases on CT imaging (Figure 6). Sensitivity for lymph node metastases is 24–43% for gastric carcinoma or 41% for carcinomas of the bladder [18,29]. In contrast, sensitivities for hematogenous metastases are 53–89% for pancreatic cancer and 33–81% for esophageal cancer [40,43,44,45]. Thus, especially the diagnosis of lymphogenous metastases is often inferior to primary tumor detection (Table 5). Most investigations on the sensitivity of CT for the identification of metastases have a different study design. The readers are usually informed about the clinical diagnosis or even that a specific type of metastasis was investigated. Hence, we assume that our sensitivity and specificity are within the normal range.

Overall, we found a high specificity for primary tumor and metastasis detection (>90%) without significant changes by the provision of clinical data. For primary tumors and lymph node metastases, specificity even increased moderately. Thus, additional clinical information does not result in overcalling in oncological imaging, also confirming previous results [5].

There are several reasons why tumors and metastases could be missed by radiologists. Firstly, the interpretation of oncological CT data sets is challenging; often multiple abnormalities are present in a single patient. Lesions may be benign and not always malignant. Therefore, findings could be undercalled and misinterpreted as benign (e.g., Figure 2) [1]. Findings may also be too small to be classified as malignant or may be overlooked (e.g., Figure 4). Additionally, findings, particularly of the lung, could be consistent with a postinflammatory benign aetiology due to configuration and subpleural location (e.g., Figure 5) [46]. Secondly, the so-called satisfaction of search represents another possible influence on the detection rate of malignant lesions. Satisfaction of search represents an interference of a radiological finding with the detection of further abnormalities [1,47]. Radiological image interpretation often follows a fixed procedure. Thus, as soon as a finding (mostly the primary tumor) is made, further lesions as metastases may be missed. This may explain the lower rate of true positive metastatic findings. Further reasons for missed metastatic lesions can be assumed. Metastases are often small, not reliably distinguishable from benign lesions and therefore an unequivocal diagnosis is often impossible without follow-up imaging or complementary procedures such as MRI, hybrid imaging, or biopsy. Moreover, imaging criteria for lymphogenous metastases are often controversial, an accurate identification is challenging. In the past, a short axis diameter of 1 cm was used as the cut-off value for enlarged, malignancy-susceptible lymph nodes [1]. Recent studies suggest different parameters for various anatomic regions, such as 6 mm for retrocrural or 8–10 mm for pelvic lymph nodes. However, enlarged lymph nodes are not always malignant. They also could have an inflammatory aetiology or normal-sized lymph nodes may have tumor involvement [48]. Of course, there are numerous cases in the present study that had been solved correctly. Figure 3 shows an example of a pancreatic carcinoma correctly diagnosed by all three radiologists irrespective of clinical information.

All these challenges in oncological imaging are reflected in interrater reliability. Across all readings, a maximum agreement rate of “moderate” was achieved. Furthermore, the influence of clinical information in primary tumor detection leads to an increase (“fair” to “moderate”), but in metastasis detection even to a decrease in the interrater reliability (“moderate” to “fair”). Studies on interrater agreement depending on additional clinical data do not exist. Thus, we are the first to show that clinical data substantially influence agreement rates of radiologists in oncological imaging. This finding might be explained by the different experience levels of the readers. Our study demonstrates a performance gap between the resident and the experienced radiologists. Without available clinical information, the experienced radiologists achieved a higher sensitivity in the detection of primary tumors, whereas readings with clinical information resulted in a lower diagnostic performance increase compared with the resident. For lymphogenous metastasis, the most experienced radiologist was inferior to both other readers when being aware of patient history. Otherwise, there were no major differences in sensitivity between the readers for metastasis detection. The resident also achieved preferable results for lymphogenous and hematogenous metastases when having clinical information. As mentioned, metastases from multiple regions in a single patient were evaluated together, resulting in a more severe rating, partially contributing to the limited detection rate.

It is a well-accepted fact that the quality of the radiological report depends on the experience level of the radiologist [2,3,4]. The majority of previous studies focused on the reading accuracy in emergency on-call imaging with mostly small differences between residents and experienced radiologists. Cooper et al. reported variances of diagnostic accuracy ranging from 1.0% to 3.3% in 141.381 patients between residents and experienced radiologists [2]. However, for more specific differential diagnoses, larger differences may occur. For example, residents’ sensitivity for suspected appendicitis in unenhanced CT in children is markedly inferior to experienced radiologists (63% vs. 95%). Specificity, in comparison, reveals no relevant difference (96% vs. 98%) [49]. Our results suggest that the experience level has a major impact on the sensitivity of the radiological report in oncological imaging.

In 2013, the European Society of Radiology (ESR) underlined the relevance of good communication between referrer and radiologist as being a key prerequisite of a high standard of care. Accordingly, radiologists should actively query clinical information from their referrers if not allocated directly. For this reason, the ESR also encourages radiologists to ensure the provision of relevant clinical information with the aim of specific and useful radiology reports, and thus benefit all, as demonstrated in this work [50]. Therefore, an awareness of the importance of clinical information in radiological reporting should be further promoted. Structured reporting has been increasingly used in clinical routines in recent years, and there are also the first positive reports on the structured recording of clinical patient data in radiology [51,52]. In the future, structured clinical data might add a pivotal value to the accuracy of radiological reporting.

Our study had several limitations. The retrospective study design with a small number of patients in each tumor entity limited the power of the diagnostic benefit from clinical information overall and for each tumor entity. Clinical information might also have elicited a significant increase in accuracy and sensitivity in experienced radiologists, which should be investigated with a larger number of patients. In addition, the study focused on the diagnosis of primary tumors. There were only a few metastases in our study population in comparison. Thus, our findings regarding the detection of metastases should not be transferred to clinical routine. Another withdrawal is that the readers were obliged to decide whether or not to record a malignant finding. Perhaps lesions were detected during the readings but due to a misinterpretation were not documented (Figure 2). In a clinical setting, these findings are usually described and have a high possibility to influence the further clinical course (e.g., follow-up imaging, biopsies, etc.). Another challenge in this study was the use of a report matrix which might influence the usual workflow, as also reported for structured reporting, and therefore generate reporting mistakes [53]. Finally, only one resident was involved as a reader, contrasted by two experienced radiologists. We showed that experienced readers clinically benefit less from information than residents. During residency training, a steep learning curve can be expected, especially during the first years [2]. Therefore, there may be a bias between the reading rounds due to a progression in the level of training. Moreover, the observation of the positive effect of clinical information on the accuracy of residents remains subject to further studies with a greater number of unexperienced readers. Ultimately, it must be considered that we only examined the performance of the individual radiologists themselves. This procedure does not correspond to the usual workflow in the clinical routine at our academic institution. Usually, the initial report is performed by a resident and the findings are reviewed by an experienced specialist. Therefore, the accuracy of radiological findings might be significantly better than the absolute values collected in this manuscript.

Overall, our results substantiate a positive impact of clinical information on radiological reporting, with a significant increase in diagnostic accuracy and sensitivity of primary tumor detection when providing clinical data. Metastasis diagnosis also benefits from clinical data, even if not significant. We found no significant change in specificity. Further studies on a larger patient cohort and number of residents are recommended to evaluate the impact of clinical information in oncological imaging. With the increasing importance of artificial intelligence in radiology, the relevance and integration of clinical information should be investigated in the future.

## 5. Conclusions

In conclusion, clinical information in oncological imaging seems to be essential for correct radiological reporting, especially for residents, and should be available for the radiologist whenever possible.

## Figures and Tables

**Table 1:** Comparison of baseline demographic features.

	Cancer Disease Present	Cancer Disease Absent	*p*-Value
Number	64	20	-
Male (%)	49 (76.6%)	12 (60%)	0.27
Median age (years) + IQR	64.5 (58–72)	58.5 (41–68.25)	0.06

**Table 2:** List of absolute and relative frequencies of the different tumor entities and locations of lymphogenous and hematogenous metastases.

**Primary Tumor**	**n = 64**
Bronchial	n = 9 (14%)
Gastroesophageal	n = 9 (14%)
Bladder	n = 9 (14%)
Renal	n = 9 (14%)
Hepatocellular	n = 9 (14%)
Pancreatic	n = 9 (14%)
Lymphoma	n = 10 (16%)
**Lymphogenous Metastasis**	**n = 22**
Thoracic	n = 8 (36%)
Abdominal	n = 11 (50%)
Iliac/Inguinal	n = 3 (14%)
**Hematogenous Metastasis**	**n = 18**
Hepatic	n = 8 (44%)
Pulmonary	n = 2 (11%)
Bone	n = 4 (22%)
Other	n = 4 (22%)

**Table 3:** Accuracy, sensitivity, and specificity of diagnostic performance for primary tumor detection depending on absence or presence of clinical information (CI).

		Accuracy		Sensitivity			Specificity		
	No CI	Given CI	*p*-Value	No CI	Given CI	*p*-Value	No CI	Given CI	*p*-Value
**Overall**	77%	87%	0.01	73%	83%	0.01	92%	98%	0.15
**Reader 1**	85%	92%	0.15	84%	91%	0.28	85%	95%	0.6
**Reader 2**	77%	83%	0.33	70%	78%	0.31	100%	100%	1.00
**Reader 3**	70%	86%	0.02	64%	81%	0.01	95%	100%	0.48
***p*-value**	0.09	0.25	-	0.03	0.14	-	0.31	1.00	-

**Table 4:** Number of correctly identified primary tumors listed per entity depending on absence or presence of clinical information (CI).

	Number of Identified Tumors per Entity								
	Reader 1		Reader 2		Reader 3		Overall		
Entity	No CI	Given CI	No CI	Given CI	No CI	Given CI	No CI	Given CI	*p*-Value
**Bronchial carcinoma**	7/9; 78%	9/9; 100%	6/9; 67%	8/9; 89%	4/9; 44%	7/9; 78%	17/27; 63%	24/27; 89%	0.05
**Gastroesophageal carcinoma**	8/9; 89%	9/9; 100%	6/9; 67%	7/9; 78%	5/9; 56%	6/9; 67%	19/27; 70%	22/27; 81%	0.53
**Renal cancer**	9/9; 100%	9/9; 100%	9/9; 100%	8/9; 89%	8/9; 89%	9/9; 100%	26/27; 96%	26/27; 96%	1.00
**Carcinoma of the bladder**	9/9; 100%	9/9; 100%	7/9; 78%	8/9; 89%	8/9; 89%	9/9; 100%	24/27; 89%	26/27; 96%	0.61
**Hepatocellular carcinoma**	7/9; 78%	7/9; 78%	6/9; 67%	5/9; 56%	7/9; 78%	7/9; 78%	20/27; 74%	19/27; 70%	1.00
**Pancreatic cancer**	7/9; 78%	8/9; 89%	5/9; 56%	7/9; 78%	5/9; 56%	7/9; 78%	17/27; 63%	22/27; 81%	0.22
**Lymphoma**	7/10; 70%	7/10; 70%	6/10; 60%	7/10; 70%	4/10; 40%	7/10; 70%	17/30; 57%	21/30; 70%	0.42

**Table 5:** Comparison of sensitivities for primary tumor detection per entity vs. reported data in the literature [11,12,13,14,15,16,17]. CI = Clinical information.

Primary Tumor	Sensitivity in the Present Study (Given CI)	Sensitivity in Literature
Bronchial	89%	98%
Gastroesophageal	81%	96%/42% ^(a)^
Bladder	96%	95%
Renal	96%	88%
Hepatocellular	70%	66%
Pancreatic	81%	76%/92% ^(b)^
Lymphoma	70%	89%

^(a)^ gastric cancer/esophageal cancer; ^(b)^ depending on size (<2 cm vs. >2 cm).

**Figure A1:**
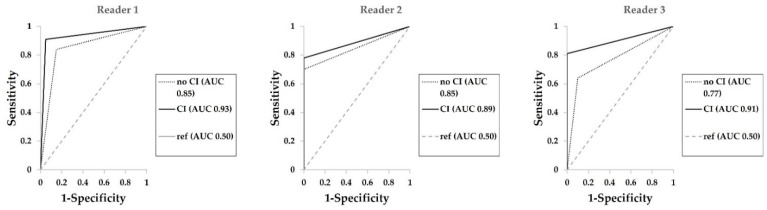
Receiver operating characteristic curves (ROC) showing the diagnostic performance of primary tumor detection for Reader 1–3 whether clinical information (CI) was provided.

## Data Availability

Not applicable.

## Appendix B

**Table A1:** True and false positive/negative findings for primary tumor detection per reader on absence or presence of clinical information (CI).

		True and False Positive/Negative Findings for Primary Tumor Detection					
		Reader 1		Reader 2		Reader 3	
		Positive to Gold Standard (n)	Negative to Gold Standard (n)	Positive to Gold Standard (n)	Negative to Gold Standard (n)	Positive to Gold Standard (n)	Negative to Gold Standard (n)
**No CI**	**Rated positive**	54 (84%)	3 (15%)	45 (70%)	0 (0%)	41 (64%)	1 (5%)
	**Rated negative**	10 (16%)	17 (85%)	19 (30%)	20 (100%)	23 (36%)	19 (95%)
**CI**	**Rated positive**	58 (91%)	1 (5%)	50 (78%)	0 (0%)	52 (81%)	0 (0%)
	**Rated negative**	6 (9%)	19 (95%)	14 (22%)	20 (100%)	12 (19%)	20 (100%)

**Table A2:** True and false positive/negative findings for lymph node metastases detection per reader on absence or presence of clinical information (CI).

		True and False Positive/Negative Findings for Lymph Node Metastasis Detection					
		Reader 1		Reader 2		Reader 3	
		Positive to Gold Standard (n)	Negative to Gold Standard (n)	Positive to Gold Standard (n)	Negative to Gold Standard (n)	Positive to Gold Standard (n)	Negative to Gold Standard (n)
**No CI**	**Rated positive**	5 (26%)	6 (9%)	11 (58%)	6 (9%)	8 (42%)	5 (8%)
	**Rated negative**	14 (74%)	59 (91%)	8 (42%)	59 (91%)	11 (58%)	60 (92%)
**CI**	**Rated positive**	5 (26%)	1 (2%)	13 (68%)	4 (6%)	14 (74%)	7 (11%)
	**Rated negative**	14 (74%)	64 (98%)	6 (32%)	61 (94%)	5 (26%)	58 (89%)

**Table A3:** True and false positive/negative findings for hematogenous metastases detection per reader on absence or presence of clinical information (CI).

		True and False Positive/Negative Findings for Hematogenous Metastasis Detection					
		Reader 1		Reader 2		Reader 3	
		Positive to Gold Standard (n)	Negative to Gold Standard (n)	Positive to Gold Standard (n)	Negative to Gold Standard (n)	Positive to Gold Standard (n)	Negative to Gold Standard (n)
**No CI**	**Rated positive**	9 (56%)	3 (4%)	9 (56%)	5 (7%)	5 (31%)	6 (9%)
	**Rated negative**	7 (44%)	65 (96%)	7 (44%)	63 (93%)	11 (69%)	62 (91%)
**CI**	**Rated positive**	9 (56%)	3 (4%)	10 (62.5%)	4 (6%)	10 (62.5%)	9 (13%)
	**Rated negative**	7 (44%)	65 (96%)	6 (37.5%)	64 (94%)	6 (37.5%)	59 (87%)

## References

[1] Siewert B., Sosna J., McNamara A., Raptopoulos V., Kruskal J.B. (2008). Missed Lesions at Abdominal Oncologic CT: Lessons Learned from Quality Assurance. Radiographics.

[2] Cooper V.F., Goodhartz L.A., Nemcek A.A., Ryu R.K. (2008). Radiology Resident Interpretations of On-Call Imaging Studies: The Incidence of Major Discrepancies. Acad. Radiol..

[3] Erly W.K., Berger W.G., Krupinski E., Seeger J.F., Guisto J.A. (2002). Radiology Resident Evaluation of Head CT Scan Orders in the Emergency Department. AJNR Am. J. Neuroradiol..

[4] Carney E., Kempf J., DeCarvalho V., Yudd A., Nosher J. (2003). Preliminary Interpretations of After-Hours CT and Sonography by Radiology Residents Versus Final Interpretations by Body Imaging Radiologists at a Level 1 Trauma Center. AJR Am. J. Roentgenol..

[5] Castillo C., Steffens T., Sim L., Caffery L. (2021). The Effect of Clinical Information on Radiology Reporting: A Systematic Review. J. Med. Radiat. Sci..

[6] Mullins M.E., Lev M.H., Schellingerhout D., Koroshetz W.J., Gonzalez R.G. (2002). Influence of Availability of Clinical History on Detection of Early Stroke Using Unenhanced CT and Diffusion-Weighted MR Imaging. AJR Am. J. Roentgenol..

[7] Doshi A.M., Huang C., Ginocchio L., Shanbhogue K., Rosenkrantz A.B. (2017). Impact of Patient Questionnaires on Completeness of Clinical Information and Identification of Causes of Pain during Outpatient Abdominopelvic CT Interpretation. Abdom. Radiol..

[8] Sarwar A., Wu J.S., Kung J., Brook A., Lee K.S., Gauguet J.-M., Rosen M.P. (2014). Graphic Representation of Clinical Symptoms: A Tool for Improving Detection of Subtle Fractures on Foot Radiographs. AJR Am. J. Roentgenol..

[9] Hopper K.D., Singapuri K., Finkel A. (2000). Body CT and Oncologic Imaging. Radiology.

[10] Wild C.P. (2019). The Global Cancer Burden: Necessity Is the Mother of Prevention. Nat. Rev. Cancer.

[11] Gould M.K., Donington J., Lynch W.R., Mazzone P.J., Midthun D.E., Naidich D.P., Wiener R.S. (2013). Evaluation of Individuals with Pulmonary Nodules: When Is It Lung Cancer? Diagnosis and Management of Lung Cancer, 3rd ed: American College of Chest Physicians Evidence-Based Clinical Practice Guidelines. Chest.

[12] Hwang S.W., Lee D.H., Lee S.H., Park Y.S., Hwang J.H., Kim J.W., Jung S.H., Kim N.Y., Kim Y.H., Lee K.H. (2010). Preoperative Staging of Gastric Cancer by Endoscopic Ultrasonography and Multidetector-Row Computed Tomography. J. Gastroenterol. Hepatol..

[13] Blick C.G.T., Nazir S.A., Mallett S., Turney B.W., Onwu N.N., Roberts I.S.D., Crew J.P., Cowan N.C. (2012). Evaluation of Diagnostic Strategies for Bladder Cancer Using Computed Tomography (CT) Urography, Flexible Cystoscopy and Voided Urine Cytology: Results for 778 Patients from a Hospital Haematuria Clinic. BJU Int..

[14] Vogel C., Ziegelmüller B., Ljungberg B., Bensalah K., Bex A., Canfield S., Giles R.H., Hora M., Kuczyk M.A., Merseburger A.S. (2019). Imaging in Suspected Renal-Cell Carcinoma: Systematic Review. Clin. Genitourin. Cancer.

[15] Roberts L.R., Sirlin C.B., Zaiem F., Almasri J., Prokop L.J., Heimbach J.K., Murad M.H., Mohammed K. (2018). Imaging for the Diagnosis of Hepatocellular Carcinoma: A Systematic Review and Meta-Analysis. Hepatology.

[16] Kitano M., Kudo M., Maekawa K., Suetomi Y., Sakamoto H., Fukuta N., Nakaoka R., Kawasaki T. (2004). Dynamic Imaging of Pancreatic Diseases by Contrast Enhanced Coded Phase Inversion Harmonic Ultrasonography. Gut.

[17] Balbo-Mussetto A., Cirillo S., Bruna R., Gueli A., Saviolo C., Petracchini M., Fornari A., Lario C.V., Gottardi D., Crescenzo A.D. (2016). Whole-Body MRI with Diffusion-Weighted Imaging: A Valuable Alternative to Contrast-Enhanced CT for Initial Staging of Aggressive Lymphoma. Clin. Radiol..

[18] Aljabery F., Lindblom G., Skoog S., Shabo I., Olsson H., Rosell J., Jahnson S. (2015). PET/CT versus Conventional CT for Detection of Lymph Node Metastases in Patients with Locally Advanced Bladder Cancer. BMC Urol..

[19] Sawczuk I.S., de Vere White R., Gold R.P., Olsson C.A. (1983). Sensitivity of Computed Tomography in Evaluation of Pelvic Lymph Node Metastases from Carcinoma of Bladder and Prostate. Urology.

[20] Pichler R., Zordo T.D., Fritz J., Kroiss A., Aigner F., Heidegger I., Virgolini I., Horninger W., Uprimny C. (2017). Pelvic Lymph Node Staging by Combined 18F-FDG-PET/CT Imaging in Bladder Cancer Prior to Radical Cystectomy. Clin. Genitourin. Cancer.

[21] Crozier J., Papa N., Perera M., Ngo B., Bolton D., Sengupta S., Lawrentschuk N. (2019). Comparative Sensitivity and Specificity of Imaging Modalities in Staging Bladder Cancer Prior to Radical Cystectomy: A Systematic Review and Meta-Analysis. World J. Urol..

[22] Zeng Y.-R., Yang Q.-H., Liu Q.-Y., Min J., Li H.-G., Liu Z.-F., Li J.-X. (2019). Dual Energy Computed Tomography for Detection of Metastatic Lymph Nodes in Patients with Hepatocellular Carcinoma. World J. Gastroenterol..

[23] Acunaş B., Rozanes I., Acunaş G., Çelik L., Sayi I., Gökmen E. (1990). Preoperative CT Staging of Colon Carcinoma (Excluding the Recto-Sigmoid Region). Eur. J. Radiol..

[24] Balthazar E., Megibow A., Hulnick D., Naidich D. (1988). Carcinoma of the Colon: Detection and Preoperative Staging by CT. AJR Am. J. Roentgenol..

[25] Zhou L., Wang J.-Z., Wang J.-T., Wu Y.-J., Chen H., Wang W.-B., Cao F., Cheng G.-X. (2017). Correlation Analysis of MR/CT on Colorectal Cancer Lymph Node Metastasis Characteristics and Prognosis. Eur. Rev. Med. Pharmacol. Sci..

[26] Rollvén E., Blomqvist L., Öistämö E., Hjern F., Csanaky G., Abraham-Nordling M. (2019). Morphological Predictors for Lymph Node Metastases on Computed Tomography in Colon Cancer. Abdom. Radiol..

[27] Dwamena B.A., Sonnad S.S., Angobaldo J.O., Wahl R.L. (1999). Metastases from Non-Small Cell Lung Cancer: Mediastinal Staging in the 1990s—Meta-Analytic Comparison Of PET and CT. Radiology.

[28] Silvestri G.A., Gonzalez A.V., Jantz M.A., Margolis M.L., Gould M.K., Tanoue L.T., Harris L.J., Detterbeck F.C. (2013). Methods for Staging Non-Small Cell Lung Cancer: Diagnosis and Management of Lung Cancer, 3rd Ed: American College of Chest Physicians Evidence-Based Clinical Practice Guidelines. Chest.

[29] Davies J., Chalmers A.G., Sue-Ling H.M., May J., Miller G.V., Martin I.G., Johnston D. (1997). Spiral Computed Tomography and Operative Staging of Gastric Carcinoma: A Comparison with Histopathological Staging. Gut.

[30] Yun M., Lim J.S., Noh S.H., Hyung W.J., Cheong J.H., Bong J.K., Cho A., Lee J.D. (2005). Lymph Node Staging of Gastric Cancer Using (18)F-FDG PET: A Comparison Study with CT. J. Nucl. Med..

[31] Nazim S.M., Ather M.H., Hafeez K., Salam B. (2011). Accuracy of Multidetector CT Scans in Staging of Renal Carcinoma. Int. J. Surg..

[32] van Vliet E.P.M., Heijenbrok-Kal M.H., Hunink M.G.M., Kuipers E.J., Siersema P.D. (2008). Staging Investigations for Oesophageal Cancer: A Meta-Analysis. Br. J. Cancer.

[33] Tseng D.S.J., van Santvoort H.C., Fegrachi S., Besselink M.G., Zuithoff N.P.A., Borel Rinkes I.H., van Leeuwen M.S., Molenaar I.Q. (2014). Diagnostic Accuracy of CT in Assessing Extra-Regional Lymphadenopathy in Pancreatic and Peri-Ampullary Cancer: A Systematic Review and Meta-Analysis. Surg. Oncol..

[34] Tseng D.S.J., Pranger B.K., van Leeuwen M.S., Pennings J.P., Brosens L.A., Mohammad N.H., de Meijer V.E., van Santvoort H.C., Erdmann J.I., Molenaar I.Q. (2021). The Role of CT in Assessment of Extraregional Lymph Node Involvement in Pancreatic and Periampullary Cancer: A Diagnostic Accuracy Study. Radiol. Imaging Cancer.

[35] Loch F.N., Asbach P., Haas M., Seeliger H., Beyer K., Schineis C., Degro C.E., Margonis G.A., Kreis M.E., Kamphues C. (2020). Accuracy of Various Criteria for Lymph Node Staging in Ductal Adenocarcinoma of the Pancreatic Head by Computed Tomography and Magnetic Resonance Imaging. World J. Surg. Oncol..

[36] Cho M.J., An C., Aljoqiman K.S., Choi J.-Y., Lim J.S., Park M.-S., Rhee H., Kim M.-J. (2020). Diagnostic Performance of Liver Imaging Reporting and Data System in Patients at Risk of Both Hepatocellular Carcinoma and Metastasis. Abdom. Radiol..

[37] Allard P., Yankaskas B.C., Fletcher R.H., Parker L.A., Halvorsen R.A. (1990). Sensitivity and Specificity of Computed Tomography for the Detection of Adrenal Metastatic Lesions among 91 Autopsied Lung Cancer Patients. Cancer.

[38] Homann G., Mustafa D.F., Ditt H., Spengler W., Kopp H.-G., Nikolaou K., Horger M. (2015). Improved Detection of Bone Metastases from Lung Cancer in the Thoracic Cage Using 5- and 1-Mm Axial Images versus a New CT Software Generating Rib Unfolding Images: Comparison with Standard 18F-FDG-PET/CT. Acad. Radiol..

[39] Lim J.S., Kim M.-J., Yun M.J., Oh Y.T., Kim J.H., Hwang H.S., Park M.-S., Cha S.-W., Lee J.D., Noh S.H. (2006). Comparison of CT and 18F-FDG PET for Detecting Peritoneal Metastasis on the Preoperative Evaluation for Gastric Carcinoma. Korean J. Radiol..

[40] Holzapfel K., Reiser-Erkan C., Fingerle A.A., Erkan M., Eiber M.J., Rummeny E.J., Friess H., Kleeff J., Gaa J. (2011). Comparison of Diffusion-Weighted MR Imaging and Multidetector-Row CT in the Detection of Liver Metastases in Patients Operated for Pancreatic Cancer. Abdom. Imaging.

[41] Loy C.T., Irwig L. (2004). Accuracy of Diagnostic Tests Read With and Without Clinical InformationA Systematic Review. JAMA.

[42] Çiray I., Åström G., Sundström C., Hagberg H., Ahlström H. (1997). Assessment of Suspected Bone Metastases: CT with and without Clinical Information Compared to CT-Guided Bone Biopsy. Acta Radiol..

[43] Tian Y., Wang C., Shan Y., Zhao D., Wang G., Zhao X., Ouyang H., Hao Y., Sun Y., Qu H. (2008). Prospective evaluation of ultrasonography, multi-slice spiral CT, endoscopic ultrasonography, and magnetic resonance imaging in assessment of TNM staging and assessment of resectability in pancreatic carcinoma. Zhonghua Yi Xue Za Zhi.

[44] Räsänen J.V., Sihvo E.I.T., Knuuti M.J., Minn H.R.I., Luostarinen M.E.S., Laippala P., Viljanen T., Salo J.A. (2003). Prospective Analysis of Accuracy of Positron Emission Tomography, Computed Tomography, and Endoscopic Ultrasonography in Staging of Adenocarcinoma of the Esophagus and the Esophagogastric Junction. Ann. Surg. Oncol..

[45] Lowe V.J., Booya F., Fletcher J.G., Nathan M., Jensen E., Mullan B., Rohren E., Wiersema M.J., Vazquez-Sequeiros E., Murray J.A. (2005). Comparison of Positron Emission Tomography, Computed Tomography, and Endoscopic Ultrasound in the Initial Staging of Patients with Esophageal Cancer. Mol. Imaging Biol..

[46] Choromańska A., Macura K.J. (2012). Evaluation of Solitary Pulmonary Nodule Detected during Computed Tomography Examination. Pol. J. Radiol..

[47] Samuel S., Kundel H.L., Nodine C.F., Toto L.C. (1995). Mechanism of Satisfaction of Search: Eye Position Recordings in the Reading of Chest Radiographs. Radiology.

[48] Ganeshalingam S., Koh D.-M. (2009). Nodal Staging. Cancer Imaging.

[49] Lowe L.H., Draud K.S., Hernanz-Schulman M., Newton M.R., Heller R.M., Stein S.M., Speroff T. (2001). Nonenhanced Limited CT in Children Suspected of Having Appendicitis: Prospective Comparison of Attending and Resident Interpretations. Radiology.

[50] (2013). European Society of Radiology (ESR) ESR Communication Guidelines for Radiologists. Insights Imaging.

[51] Granata V., Pradella S., Cozzi D., Fusco R., Faggioni L., Coppola F., Grassi R., Maggialetti N., Buccicardi D., Lacasella G.V. (2021). Computed Tomography Structured Reporting in the Staging of Lymphoma: A Delphi Consensus Proposal. J. Clin. Med..

[52] Granata V., Caruso D., Grassi R., Cappabianca S., Reginelli A., Rizzati R., Masselli G., Golfieri R., Rengo M., Regge D. (2021). Structured Reporting of Rectal Cancer Staging and Restaging: A Consensus Proposal. Cancers.

[53] dos Santos D.P., Hempel J.-M., Mildenberger P., Klöckner R., Persigehl T. (2019). Structured Reporting in Clinical Routine. Rofo.

